# Glycosphingolipids as Receptors for Non-Enveloped Viruses

**DOI:** 10.3390/v2041011

**Published:** 2010-04-15

**Authors:** Stefan Taube, Mengxi Jiang, Christiane E. Wobus

**Affiliations:** Department of Microbiology and Immunology, University of Michigan Medical School, 5622 Medical Sciences Bldg. II, 1150 West Medical Center Dr., Ann Arbor, MI 48109, USA; E-Mails: stau@umich.edu (S.T.); jiangm@umich.edu (M.J.)

**Keywords:** non-enveloped virus, glycosphingolipid, receptor, calicivirus, rotavirus, polyomavirus, parvovirus

## Abstract

Glycosphingolipids are ubiquitous molecules composed of a lipid and a carbohydrate moiety. Their main functions are as antigen/toxin receptors, in cell adhesion/recognition processes, or initiation/modulation of signal transduction pathways. Microbes take advantage of the different carbohydrate structures displayed on a specific cell surface for attachment during infection. For some viruses, such as the polyomaviruses, binding to gangliosides determines the internalization pathway into cells. For others, the interaction between microbe and carbohydrate can be a critical determinant for host susceptibility. In this review, we summarize the role of glycosphingolipids as receptors for members of the non-enveloped calici-, rota-, polyoma- and parvovirus families.

## 1. Introduction

Viruses come in many different flavors and are often broadly classified based on their nucleic acid content (DNA *versus* RNA virus), capsid symmetry (icosahedral, helical, or complex), and the presence or absence of a lipid envelope (enveloped *versus* non-enveloped). Each of these characteristics results in specific replication strategies. However, all viruses are obligate intracellular pathogens that rely on the cellular machinery for all stages of the life cycle [1]. Virus infection of cells is a multi-step process that generally can be divided into: i) binding to cell surface receptors, ii) fusion with the plasma membrane (some enveloped viruses) or internalization and membrane penetration from intracellular compartments (enveloped and non-enveloped viruses), iii) trafficking to the site of replication and/or uncoating of the viral genome, iv) replication, iv) production of virion progeny, and v) egress. Virus binding to the cell surface is the first step during infection and therefore a determinant for tropism. This interaction is also an attractive target for antiviral therapy. Viruses can bind many different molecules, including proteins, lipids, and carbohydrates. Some are attachment factors, which concentrate virus on the cell surface, while others are receptors or co-receptors that facilitate virus entry into cells. Viruses can also use alternate receptors depending on the cell type. Molecules on the cell surface available for virus binding are typically glycoconjugates (e.g., glycosphingolipids, glycoproteins, and proteoglycans) [2]. While the glycan portion usually extends furthest into the extracellular space, virus binding cannot only occur to the membrane distal saccharide but also to the membrane proximal protein core. Binding to glycans is in many cases mediated by electrostatic forces to negatively charged sialic acid-containing oligosaccharides or portions of glycosaminoglycans. In some cases, the same oligosaccharide motif can be present on both glycolipids and glycoproteins, e.g., histo-blood group antigens (HBGAs). In this review, we will specifically focus on binding of non-enveloped viruses to glycosphingolipids.

## 2. Discussion

### 2.1. Structure and function of glycosphingolipids

Glycosphingolipids (GSLs) are sphingosine-containing glycolipids [3]. These amphiphatic molecules made up of hydrophobic ceramides (e.g., sphingosine) and hydrophilic carbohydrates are integral parts of most lipid bilayers together with phospholipids and cholesterol [3]. The chemical make-up of these basic building blocks of all living cells contributes to local membrane curvature because of the large bulky carbohydrate head group compared to the lipid. The longer the carbohydrate chain and the larger the segregation, the more pronounced the membrane curvature [4]. Furthermore, the carbohydrates of GSLs are typically aligned perpendicular to the lipids, *i.e.,* lie on the membrane. This is contrary to glycoproteins, which extend their carbohydrate chains into the extracellular space. Therefore, only the outer surface of the carbohydrate chain is accessible for interactions with ligands, e.g., antibodies, lectins, and other GSLs.

**Figure 1.**
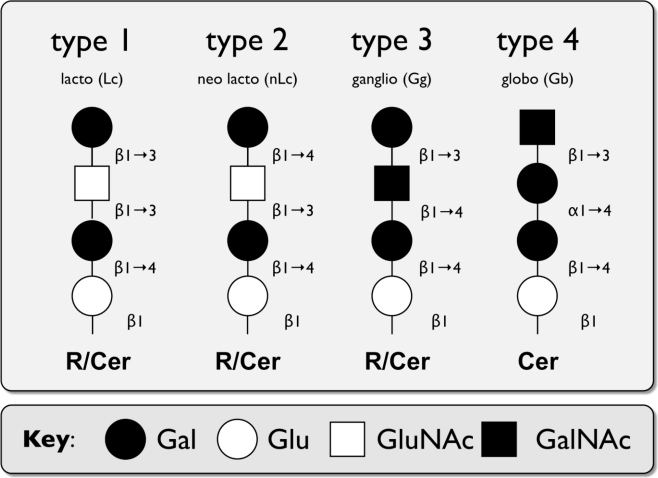
Schematic representation of discussed major core structures of vertebrate glycosphingolipids (GSLs) (based on IUPAC-IUB Joint Commission on Biochemical Nomenclature [6]). Abbreviations: Glu: glucose, Gal: galactose, GluNAc: N-acetylglucosamine, GalNAc: N-acetylgalactosamine, Cer: ceramide, R: residue

The wide variety of the different GSLs structures is mainly due to differences in number and type of the carbohydrate building blocks and/or the length, saturation status, and hydroxylation level of the fatty acid chain. GSLs are typically classified based on their charges (neutral, acidic, basic) or core structures (lacto-, gala/neolacto-, ganglio-, globo-series) [3,5] (Figure 1). Acidic GSLs contain sialic acid or sulfate groups, while basic GSLs are rare. Gangliosides, *i.e.,* GSLs with sialic acid in the carbohydrate chain, are found in all series and are made up of ceramide and mono- or oligosaccharides [3].

GSLs are synthesized in the endoplasmic reticulum (ER) and Golgi apparatus via sequential addition of carbohydrate units by specific glycosyltransferases and are then transported to the cell surface [7]. At the plasma membrane the carbohydrate moiety is exposed to the extracellular space. GSLs are also associated with intermediate filaments within the cell but this may represent an intracellular transport intermediate [7]. On the cell surface, GSLs are differentially expressed in different cell types, tissues, and species. Their expression is normally tightly regulated during development and differentiation, but when dysregulation occurs GSLs can also be tumor-associated antigens [8]. Accumulation of GSLs can lead to debilitating diseases such as Tay-Sachs disease, Gaucher’s disease or Krabbe’s disease [3]. Cell surface expression of GSLs can also be modified by hydrolysis via membrane-bound sialidases on the cell surface of the same or neighboring cell or by shedding of GSL-enriched vesicles. Collectively this leads to changes in GSL composition and organization that may have biological consequences with regards to membrane curvature or function (e.g., modulating activity of associated proteins or their signaling capabilities).

GSLs play many important functional roles in cell adhesion and cell signaling based on their preferential distribution in the outer leaflet of the lipid bilayer on the cell surface [3]. They mediate cell adhesion by interaction with lectins (carbohydrate binding proteins), e.g., selectins, siglecs, or by carbohydrate-carbohydrate interaction. Their role in signaling is two-fold, either as modulators of signal transduction through association with signal transduction molecules in lipid microdomains (e.g., lipid rafts), or the direct interaction (inhibition or stimulation) with growth factors or integrin receptors [3,4,8]. In fact, GSL-enriched microdomains in the plasma membrane are structural units that mediate GSL-dependent cell adhesion and signal transduction. GSLs order biological membranes to form lipid membrane microdomains. The ceramide portion is particularly important for this clustering in microdomains. GSLs can associate with a pre-existing ordered domain or segregate into their own. This results in GSL-enriched microdomains that have different lipid compositions from other membrane regions involved in many cellular events. While part of these microdomains, GSLs can influence the function of membrane-associated proteins because the sphingolipids in these microdomains restrict fluidity; thus, confining membrane-associated proteins, favoring lateral interactions between proteins within a domain, or preventing interactions with proteins in different domains. On example of the former is the formation of a complex between the tetraspanin CD9, GM3, integrin α3 or α5 that results in reduced cell motility. Furthermore, proteins in GSL-rich areas tend to interacts laterally with confining lipids. For example, the “glycosynapse” is a membrane microdomain involved in carbohydrate-dependent adhesion through GSL-GSL interactions or GSL-dependent modulation of adhesion receptors like integrins.

GSLs also directly function as receptors for cellular molecules (hormones, interferons and lymphokines) or microbes (bacteria, viruses) and microbial products (bacterial toxins) via their carbohydrate structure [8,9]. While many different microbes bind to GSLs, a few common features of these interactions are seen repeatedly [9]. Terminal or internal (non-terminal) portions of the GSL can function as the minimal binding site. Shorter GSLs with binding epitopes in close proximity to the lipid bilayer are utilized more frequently as receptors, often facilitating internalization. A few changes in the amino acid sequence of closely related strains/variants in or around the receptor binding site can lead to differences in binding specificities. Carbohydrate-microbe interactions are typically of low affinity and are strengthened by multivalency of repeating units (e.g., icosahedral virions, repeating saccharide units). One or more of these features are found in all microbe-GSL interactions, including the interaction of non-enveloped viruses with GSLs, which is the topic of this review.

### 2.2. Caliciviridae

Caliciviruses are small non-enveloped icosahedral viruses with a single-stranded, plus-sense RNA genome of 7.4–8.3 kb [10]. They are divided into the genera *Norovirus* (NoV), *Sapovirus* (SaV), *Vesivirus* (VeV), *Lagovirus* (LaV), and *Nebovirus* (NeV), as well as the recently proposed “*Recovirus”* (ReV) [10–12]. Caliciviruses in general exhibit a wide range of host and tissue tropisms, causing a variety of diseases [10]. Only members of the NoV and SaV genera infect humans causing gastroenteritis. VeV includes the feline calicivirus (FCV), which causes respiratory disease in cats. LaV includes rabbit hemorrhagic disease virus (RHDV) that causes a necrotizing hepatitis with associated hemorrhaging and high mortality rates in rabbits. NeV and “*Recovirus*” have only recently been described and include bovine enteric virus Newbury agent-1 (Newbury1) and the rhesus macaques Tulane virus (TV), respectively.

The genomes are typically organized into either two (LaV, SaV, NeV) or three (NoV, VeV, ReV) major open reading frames (ORF) [10–12]. ORF-1 encodes a non-structural polyprotein processed by the virally encoded 3C-like cysteine protease. ORF-2 encodes the major capsid protein VP1 that forms the viral capsid [10]. ORF-3 encodes the minor capsid protein VP2, of which 1-2 copies are incorporated into the capsid [13]. A fourth ORF was recently identified by bioinformatics for murine norovirus (MNV) [14]. In the case of SaV, LaV, and NeV, VP1 is part of the ORF-1 polyprotein [10].

The viral capsid is composed of 180 monomers of the major capsid protein VP1, which is organized into 90 dimers and exhibits a T=3 icosahedral symmetry [15–19]. The capsid contains two domains, the shell (S) and the protruding (P) domains, which are separated by a flexible hinge [15–17,20]. The S domain forms a smooth shell around the viral genome, but is unable to bind to receptors [21,22]. The P domain contains receptor-binding regions and dimerizes forming arch-like structures on the capsid surface [23–27]. It is further subdivided into P1 (stem of arch) and P2 (top of arch) subdomains. P2 is the most variable region and contains the carbohydrate-binding motif [25–27]. Expression of VP1 in various insect, animal, or plant expression systems results in formation of virus-like particles (VLPs) that antigenically and morphologically resemble infectious virus [28–33].

The study of calicivirus biology is hampered by the lack of efficient cell culture systems and only MNV [34], the SaV porcine enteric calicivirus (PEC) [35], and FCV [36] are efficiently cultured in continuous cell lines. Based on studies in culture as well as using VLPs, it appears that a common feature of caliciviruses is their ability to attach to polymorphic glycoconjugates (GSL, glycoproteins or glycosaminoglycans). Many human noroviruses (HuNoV) and RHDV bind to HBGAs, while MNV-1 attaches to sialic acid on gangliosides (see below). Furthermore, the F9 strain of FCV attaches to α2,6-linked sialic acid on N-linked glycoproteins [37] while some other FCV isolates can infect cells independently of α2,6-linked sialic acid [38]. Entry of FCV is then mediated by the feline junctional adhesion molecule A (fJAM-A), a transmembrane Ig-like protein [39]. Thus far fJAM-A constitutes the only known functional receptor in the calicivirus family. Virus entry into permissive cells then occurs either by pH-dependent, clathrin-mediated endocytosis for FCV [40] or a pH-independent, cholesterol- and dynamin-dependent endocytosis in the case of MNV [41–43].

#### 2.2.1. Noroviruses (NoV)

NoV are emerging pathogens [44–47] that have been isolated from an increasing number of animal species, including mice, cows, pigs, and sheep [48–52]. NoV are classified into five different genogroups (GI-GV) and at least 40 different genotypes [53,54]. Members of GI, GII and GIV infect humans, whereas GIII infects only bovine species [53] and GV only mice [48]. Currently, HuNoV strains belonging to genogroup II genotype 4 (GII.4) are the predominant outbreak strains worldwide (e.g., [55–57]). Early steps in the norovirus life cycle (e.g., receptor binding) are determinants of norovirus tropism [58] and thereby determine the outcome of a viral infection.

##### 2.2.1.1. Human noroviruses (HuNoV)

HuNoV are responsible for the majority of acute viral gastroenteritis worldwide with an estimated 23 million infections in the US per year [10,59]. HuNoV infections affect all age groups and transmission occurs through contaminated food or water and person-to-person contact via the fecal-oral route or virus-containing aerosols [10]. Outbreaks frequently occur in enclosed facilities, such as hospitals, nursing homes, and cruise ships. Although infections are usually mild and non life-threatening in healthy adults, they pose a serious threat to the immunocompromised and elderly. An important complication of norovirus containment is the spread of virus by asymptomatically infected people as well as patients, who have recovered from disease symptoms, but are still shedding virus. However, despite their impact on public health, no drugs or vaccines are available for treatment or prevention [10].

Despite the inability of HuNoV to grow reproducibly in cultured cells [60], the availability of VLPs has enabled binding studies and the identification of attachment receptors. These VLPs are generated by spontaneous self-assembly of the capsid protein VP1, which morphologically and antigenically resemble native virions [29]. Most HuNoV bind to HBGAs (reviewed in: [44,61,62]), while some can also bind to heparan sulfate [63] or sialic acid [64] (Table 1).

HBGAs consist of complex glycans and are found in a variety of cell types, including red blood cells [65] and intestinal epithelial cells [66]. They can also be secreted into body fluids, such as breast milk and saliva [67,68]. HBGAs are generated by an ordered addition of monosaccharides by glycan modifying enzymes. The antigens producing polymorphic ABH, Lewis, and secretor phenotypes can be found on a variety of N- and O-linked glycoproteins, as well as GSLs of the lacto-, neolacto-, ganglio-, and globoseries [6,67,69] (see Table 2 and Figure 1).

**Figure 2.**
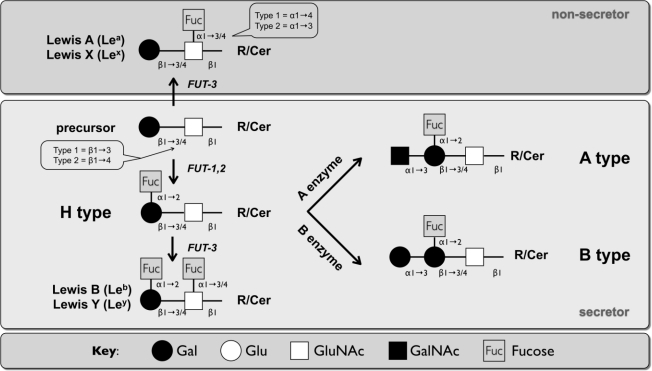
The biosynthetic pathway of type 1 (Lc) and type 2 (nLc) HBGAs. The pathway starts with a disaccharide precursor of type1 or type 2 carbohydrate chains. Type 1 disaccharide precursors are β1-3, and type 2 are β1-4 linked. FUT1 specifically generates type 2 H antigens, while FUT2 preferentially produces the type 1 H antigens, although it also has activity on the type 2 chain [109]. Further modification of the H type1/2 by A or B enzymes synthesizes the respective tetrasaccharides, adding the A and B antigens. FUT3 generates the trisaccharides Lewis A (Le^A^) or Lewis X (Le^X^) in non-secretors, as well as Le^B^ and Le^Y^ in secretors, by attaching α1-4 fucose on type 1 chains or α1-3 fucose on type 2 chains. Abbreviations: Glu: glucose, Gal: galactose, GluNAc: N-acetylglucosamine, GalNAc: N-acetylgalactosamine, Cer: ceramide, R: residue

The biosynthetic pathway of HBGAs involves the linkage of glycans and oligosaccharide chains to a disaccharide precursor [5,8,99] (Figure 2). Addition of fucose by an alpha-1,2-fucosyltransferase generates the H antigens, which can be followed by the biosynthesis of the A or B antigens through the activities of the blood group A or B transferases, respectively. There are two functional alpha-1,2-fucosyltransferases known in humans, encoded by two genes, *FUT1* and *FUT2*. FUT1 is preferentially expressed in ecto- and mesodermally derived tissue (e.g., red blood cells), producing mostly type 2 antigens, whereas FUT2 based type 1 antigens are found in endodermally derived tissue (e.g., epithelial cells in the intestinal tract) [100]. Individuals that lack FUT1 produce the rare Bombay phenotype, characterized by the lack of ABH antigens on red blood cells [101]. Individuals that lack FUT2 produce the non-secretor phenotype (about 20% of Caucasians) and are characterized by the absence of ABH antigens in body fluids and various epithelial cells [68]. FUT3 is an alpha-1,4-fucosyltransferase and produces the Lewis antigens Le^A^ (type 1) and Le^X^ (type2) in non-secretors and Le^B^ (type 1) and Le^Y^ (type 2) in secretors. A-type epitopes are also found on type 3 (ganglio) and type 4 (globo) oligosaccharide chains [65].

**Figure 3.**
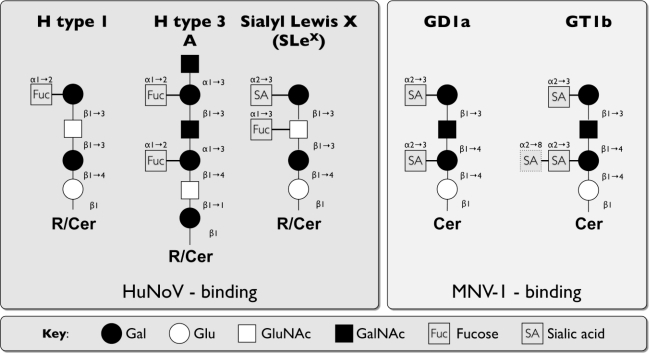
Comparison of some discussed carbohydrate structures used during attachment of human and mouse NoV. Schematic representation of the HuNoV attachment receptors (H type 1, H type 3 A, Sialyl Lewis X) and MNV-1 ganglioside attachment receptors (GD1a and GT1b). Abbreviations: Glu: glucose, Gal: galactose, GluNAc: N-acetylglucosamine, GalNAc: N-acetylgalactosamine, Cer: ceramide, R: residue

Noroviruses recognize polymorphic HBGAs on type 1, 2 and 3 GSL as attachment receptors [102.^106^] via the P2 subdomain of VP1 [21, 25–27]. HBGA-binding is diverse and strain-specific, as some strains bind exclusively to either ABH or Lewis, both, or neither (e.g., [102,105,107,108]). This was demonstrated in multiple studies using VLPs from a variety of NoV strains and testing VLP binding to saliva from secretors or non-secretors or individuals with different ABH and Lewis profiles as well as synthetic oligosaccharide chains containing human HBGA epitopes. However, these structures can be present on either lipids or proteins (see Table 1) and only a few recent studies have investigated NoV binding to carbohydrates in complex with its core. Binding of Norwalk virus (NV) to GSLs was first demonstrated using radioactively-labeled VLPs in thin-layer chromatogram (TLC) binding assays [109]. NV VLPs bind to all type 1 and 2 secretor epitopes but not B-types, as well as complex GSLs on blood group O and A but not B erythrocytes. Furthermore, studies using quartz crystal microbalance with dissipation (QCM-D) monitoring and TLC show that NV and Dijon (GII.4) attach to the H type 1 antigen on GSL but not Le^A^ [110]. Interestingly, a threshold concentration of H type 1 GSL was required for binding of both strains. However, the threshold of Dijon binding was one order of magnitude higher than that of the NV strain, indicating a lower affinity of Dijon to H type 1 GSLs. The authors propose that binding of virus to a few GSL molecules recruits further molecules into the binding area to increase affinity of the interaction [110]. Furthermore, one study compared a large panel of VLPs from five GI genotypes and eight GII genotypes for binding to ABH and Lewis HBGAs attached to type1 or type 2 core structures by ELISA and Biacore analysis [111]. Several VLPs were identified that showed slower dissociation rates in the Biacore experiments and stronger binding of VLPs in ELISA to type 1 carbohydrates compared to type 2 carbohydrates; thus demonstrating that core structures are also important for VLP recognition. This is partially consistent with the previous observation that NV binds type-1 and -3 HBGA (but not type 2) on gastroduodenal epithelial cells of secretor individuals [106]. However, the relevance of this differential recognition during norovirus pathogenesis remains unknown. In addition, a recent study also identified binding of HuNoV VLPs to negatively charged sialylated neoglycoproteins [64] instead of neutral HBGAs. Since the same sialylated structures are also present on GSL, this raises the possibility that HuNoV can also bind to acidic GSL. VLPs from the Chron1 (GII.3) and the Dijon strains recognize sialyl Lewis x (SLe^X^) and the structural analogues sialyl-diLewis x (2SLe^X^) and sialylated type 2 antigen in addition to secretor gene-dependent glycans (Figure 3) [64]. Binding to SLe^X^ is strictly sialic acid-dependent, since a non-sialylated control glycan (Lex-^HSA^) does not bind to these VLPs. Also, both VLPs do not bind to SLe^A^ indicating that the fucosyl residue of SLe^X^ is not absolutely required and that a terminal sialic acid is not sufficient for binding. In contrast, NV VLPs do not recognize any of the sialylated conjugates and only recognized secretor gene-dependent glycans on GSL [64]. So far no GI strains have been described to bind sialylated antigens [54].

Furthermore, epidemiological and challenge studies also reveal a connection between HBGA expression, secretor status and susceptibility to HuNoV strains [61,105,108,112,113]. For example, a challenge study with NV showed that non-secretor individuals are resistant to infection [105], and NV VLPs bind to gastroduodenal cells from secretors but not non-secretors [106]. In addition, NV VLPs hemagglutinate erythrocytes and bind to saliva from blood group A, AB, and O individuals, but less from blood group B [102,104,114]. Non-secretors are typically less likely to be infected [115,116]. However, this is not universal as VLPs from an epidemic GII.4 strain from 2002 (Farmington Hills) bind to saliva from both secretors and non-secretors [117]. Studies using a GII.2 strain (Snow Mountain) showed that blood type and secretor status did not correlate with infection [116] and a group of genetically susceptible individuals never got infected [105], suggesting that additional factors may exist that determine HuNoV susceptibility.

Analysis of crystal structures of the P domains from strains in two genogroups NV (GI.1) or VA387 (GII.4) in complex with oligosaccharides from HBGA reveals two distinct and spatially separated carbohydrate binding sites in the P2 subdomain [25–27]. NV and VA387 bind the same ligand (A trisaccharide) using two independent sites and binding modes [25–27]. NV binds to synthetic H type and A type carbohydrates via hydrogen bonds from a binding pocket at the interface of the P-dimer [25,26], while VA387 binds to the A and B antigen via hydrogen bonds to specific amino acids in the E’F’-loop in each P domain monomer [27]. NV VLP binding to the H type pentasaccharide is mediated primarily via the α-fucose and β-galactose groups [26] while α-fucose is less important in case of A type trisaccharide binding and most hydrogen bonds are with the αGalNAc group [25,26]. Binding of the A type trisaccharide is mediated primarily through hydrogen bonding to the α-fucose group with weaker, long-distance interactions to the β-galactose ring. Some amino acids in the E’F’-loop in the VA387 structure could only be resolved in the complexed structure, indicating a degree of flexibility in the binding pocket that becomes stabilized upon receptor binding [27]. Additionally, the same amino acids in the NV binding pocket can interact with different molecules in the oligosaccharide chain. Alpha-1,2-fucose is preferentially bound in the H type, while the same amino acids interact with the terminal GalNAc in the A type structure. A superimposition of the GII.4 structure (VA387) with modeled structures of other GII P domains showed that the binding site differs not just between genogroups, but also between different genotypes within one genogroup [118].

Variations in and around the HBGA binding domain result in altered HBGA binding and antigenicity and may be a mechanism by which noroviruses escape immune response and herd immunity while retaining receptor binding activity [118]. For instance, a single amino acid exchange at position 395 in the P2 domain of the Grimsby strain (secretor-dependent) allows the virus to infect non-secretors and thereby expand into a naïve population [118]. Additional changes that alter antigenicity then allow the virus to escape herd immunity [54,117,118]. Thus, new progeny variants are capable of infecting the same individuals that were previously infected or previously resistant individuals.

##### 2.2.1.2. Murine Norovirus

**Figure 4.**
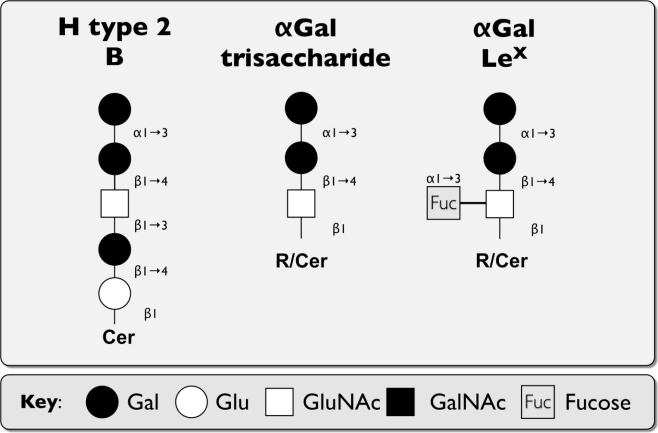
Comparison of discussed carbohydrate structures used during attachment of bovine NoV. Schematic representation of the BoNoV attachment receptor (αGal and αGal-Le^X^) in comparison to the human HBGA receptor H type 2 B-antigen. Abbreviations: Glu: glucose, Gal: galactose, GluNAc: N-acetylglucosamine, GalNAc: N-acetylgalactosamine, Cer: ceramide, R: residue

The first murine norovirus (MNV) strain, MNV-1, was discovered in 2003 and was the first member of GV [48]. Serologic analysis of mice from research colonies in North America, Europe and Asia demonstrated that 22–64% of all mice had antibodies against MNV-1 or were positive for MNV genome, which makes MNV the most prevalent virus in research mice [119–124]. MNV shares pathogenic properties with HuNoV as it is an enteric virus that replicates in the intestine and is shed in feces, resulting in fecal-oral spread [125]. While the tropism for HuNoV remains unknown, MNV efficiently replicates in murine macrophages and DCs, but not in human macrophages and DCs or other cell types regardless of their origin [34,125]. MNV-1 enters permissive murine macrophages and DCs in a pH-independent manner [42]. MNV-1 does not bind to commercially available synthetic HBGAs [54]. Instead, binding of MNV-1 to the macrophage cell surface is partially neuraminidase-sensitive and ganglioside-dependent [70]. TLC demonstrated that murine macrophages express the gangliosides GD1a, GM1, GA1 (Figure 4) and MNV-1 binds to GD1a, but not GM1 and GA1 by ELISA [70]. This suggests that the minimal binding epitope is the terminal sialic acid found in GD1a. The same terminal sialic acid is also found in GT1b and MNV-1 binds to GT1b by ELISA verifying this observation (Taube, S. and Wobus, C. E. unpublished observation) (Figure 3, Table 1). Ganglioside depletion and reconstitution studies with GD1a and GA1 demonstrate that MNV-1 and two other MNV strains (WU11 and S99) bind specifically to GD1a on murine macrophages [70]. In summary, data from both HuNoV and MNV highlight the use of carbohydrates during binding using at times the same side group, *i.e.,* usage of sialic acids (MNV-1, HuNoV strains Chron1 and Dijon), or similar carbohydrate backbones, *i.e.,* Gal-GalNAc-Gal sequence in GD1a/GT1b and H type 3 (Figure 3).

##### 2.2.1.3. Bovine Norovirus (BoNoV)

The bovine norovirus Newbury2 (NB2) (Bo/Newbury2/1976/UK) was first isolated in the UK in 1976 from calves with diarrhea and is the type strain of GIII [71,126]. Labeling of bovine gut sections with VLPs from NB2 matched the staining pattern of the αGal epitope detected by either a mAb or the GS1-B4 lectin, but not that of ABH or Lewis antigens and was sensitive to α-galactosidase treatment [71]. Using a wide range of synthetic oligosaccharides, the αGal epitope reacted strongest with NB2 VLPs, with αGal-Le^X^ being the only other oligosaccharide detectable by ELISA. Saturation transfer difference (STD) nuclear magnetic resonance (NMR), which allows identification and characterization of ligand binding to large receptor proteins at an atomic resolution, identified the α1,3-glycosidic linkage of αGal as the central recognition element of Galα3GalβOMe in binding to NB2 VLPs [71] (Figure 4). Even though the αGal epitope is structurally related to the H type 2 B-antigen, NB2 is not likely to infect humans, because the αGal epitope is absent from human duodenal cells, and NB2 does not hemagglutinate human B blood group erythrocytes [71].

#### 2.2.2. Lagovirus

Rabbit Hemorrhagic Disease Virus (RHDV), the type species in the genus LaV, was the first calicivirus shown to bind HBGA [72]. It causes systemic infection in rabbits with epidemics of acute fatal hepatitis, but the mechanism by which RHDV infects rabbits is still unclear [72,127]. RHDV replicates in hepatocytes and large numbers of virus are found in liver tissue from infected animals [128,129]. However, recombinant VLPs or virions fail to bind to liver sections but instead bind to HBGA on rabbit epithelial cells of the upper respiratory and digestive tracts [72,130].

RHDV hemagglutinates human and rabbit red blood cells [72,128,129]. Hemagglutination is dependent on the presence of ABH blood group antigens, because the rare Bombay phenotype, which completely lack ABH antigens, is not agglutinated [72]. Furthermore, agglutination was inhibited by saliva from secretor individuals, but not from non-secretors [72]. RHDV VLPs and virions from liver extracts specifically bind synthetic A and H type 2 blood group oligosaccharides, but not Le^B^ or Le^Y^ antigens [72]. The L-fucose is the minimal structural epitope required for RHDV VLP binding because VLPs very selectively recognized α-L-Fuc-OMe but not β-D-Gal-OMe by STD-NMR [131].

Similarly to human secretor status, nonfunctional alleles of *FUT* genes confer an important genetic resistance marker to RHDV infection. Generation of the H type 2 HBGA requires an alpha-1,2 fucosyltransferase [72]. In rabbits three alpha-1,2 fucosyltransferase genes are known, *Fut1*, *Fut2*, and *Sec1*. Nonfunctional alleles in any of these loci are known to occur [127]. Mutations leading to the H type 2-negative phenotype are predominant in areas with a high impact of RHDV as demonstrated by genotyping buccal epithelial cells from wild rabbits [127]. In addition, age-dependent expression of H type 2 correlates with an increase in susceptibility RHDV infection [72]. Young rabbits are protected from infection, and their tracheal tissues were almost devoid of A and H type 2 antigens with only very weak binding of VLPs [132,133]. Since HBGA are undetectable on rabbit hepatocytes, it is unlikely that RHDV uses HBGAs as receptors on hepatocytes. Instead HBGAs likely mediate infection of upper respiratory and/or digestive tract epithelial cells. However, whether HBGAs constitute a functional receptor or attachment factor remains to be investigated.

### 2.3. Rotaviruses

Rotaviruses are members of the *Reoviridae* family [134]. They are the leading cause of gastroenteritis in children worldwide and are important veterinary pathogens (e.g., cattle, sheep, swine, and poultry). Rotavirus infections in nature occur mostly in a species-specific manner. The virus targets the mature enterocytes at the tip of the intestinal villi in the small intestine of all ages but disease is primarily observed in the young. The watery diarrhea is caused by multiple factors that include malabsorption and a secretory component [135].

The rotavirus genome is composed of 11 segments of double-stranded RNA, which are enclosed in triple-layered particles of approximately 100 nm in diameter [73]. The inner capsid layer is formed by VP2 and encloses a less well-defined subcore that also contains the VP1 (RNA-dependent RNA polymerase) and VP3 (guanylyltransferase and methylase), both of which form a complex at the icosahedral five-fold vertices. The intermediate layer is made up of VP6, and the outer layer consists of 780 molecules of the glycoprotein VP7 and 60 dimers of spikes of VP4 [73,134]. Both VP7 and VP4 interact with cellular receptors and facilitate virus entry into cells. Virus entry is a multi-step process that depends on multiple cellular molecules: N-acetylneuraminic (sialic) acid residues, integrins α2β1, α4β1, αvβ3, αxβ2, heat shock cognate protein (hsc) 70, and certain gangliosides [73,136] (Table 1). Trypsin cleavage of VP4 leads to conformational changes in the capsid and is required for virus entry (but not binding). The cleavage yields the C-terminal VP5* domain of VP4 and a N-terminal VP8* domain of VP4, both of which remain associated with the particle. A sequential model was proposed for the early steps of rotavirus entry into cells [134]. First, the VP8* domain of VP4 interacts with sialic acid residues (either on glycosphingolipids or glycoproteins) that induces a conformational change in VP4, exposing VP5*. Second, the VP5* domain interacts with α2β1 integrin. Third, the following interactions occur in a yet to be determined sequence of events sequentially or alternatively: VP5* with hsc70, VP5* with α2β1, VP5* with α4β1, VP7 with αvβ3 integrin, and (depending on the strain) VP7 with αxβ2 or α4β1 integrins. However, α2β1 integrin is not necessary for attachment to cells [137]. Interaction with these multiple receptors occurs at least in part in lipid raft microdomains [134]. VP8* causes an opening of the tight junctions, which may be required in polarized epithelial cells, which express integrins primarily on the basolateral surface. Based mostly on studies in non-polarized epithelial African green monkey kidney cells (MA104) with the rhesus rotavirus (RRV) strain, virus entry then occurs in a caveolin-, clathrin-independent but dynamin- and cholesterol-dependent manner. Plasma membrane penetration is then mediated by VP5*, which binds lipids and permeabilizes membranes [138,139]. Once the virions reach the cytoplasm, the triple-layer particle sheds its outer layer to yield the transcriptionally active double-layer particle and viral replication proceeds in the cytoplasm.

For the purpose of this review, we will focus only on the interaction of rotavirus (via VP8*) with its glycosphingolipid attachment receptor. The recognition that sialic acids play an important role during rotavirus entry comes from early studies in which some strains had hemagglutination activity of red blood cells and neuraminidase treatment of cells reduced virus binding and infection of some strains (e.g., [140–144]). In general, the easily culturable animal rotaviruses bound to host cells in a neuraminidase-sensitive manner while human rotaviruses did not [145]. This led to the classification as “neuraminidase-insensitive = sialic acid-independent” *versus* neuraminidase-sensitive = sialic acid-dependent” rotaviruses. This controversial classification of sialic acid-dependent and –independent rotaviruses strains has now been laid to rest as recent structural data [76] provides conclusive evidence that neuraminidase-insensitive viruses bind to internal sialic acids resistant to the action of neuraminidases. Therefore, all rotaviruses are thought to bind to sialic acids but the relative position and/or linkage of the sialic acid residue may differ [146]. Strains that dependent on terminal sialic acid as binding partners also typically hemagglutinate erythrocytes. However, viruses from either group infect polarized cells through either the apical or basolateral membrane with a slight preference for the latter [147]. This likely contributes to the observed extraintestinal spread of rotaviruses [148,149]. Furthermore, important for rotavirus pathogenesis is the finding that rotavirus-binding competent gangliosides are expressed in tissues in an age-dependent manner, which is one likely reason for the observed age-dependent disease manifestation.

#### 2.3.1. Neuraminidase-sensitive strains

##### 2.3.1.1. Porcine rotaviruses (OSU, CRW strains)

Porcine rotaviruses are important agricultural agents that bind to cells in a neuraminidase-dependent manner [74,75,150–152]. Purified GM2 and GM3 have the greatest inhibitory effect in preventing binding of iodinated triple-layered OSU particles to porcine enterocytes and MA104 cells (embryonic rhesus monkey kidney cells) [74,153]. Some inhibition was seen with GM1 and GD1a, even less with GD1b and GT1b and no inhibition with GA1 and GA2 [74,153]. To correlate these findings to pathogenesis, GSLs were isolated from pooled intestines of newborn to four week-old piglets and porcine GM3 (either N-glycolylneuraminic acid [NeuGc] or N-acetylneuraminic acid [NeuAc]) was identified as the attachment receptor of OSU [74,75]. *In vitro*, iodinated triple-layered but not double-layered particles bind to intestinal NeuAcGM3, NeuGcGM3, and bovine GM3 immobilized on TLC plates. In culture, intestinal GM3 blocks binding and infection of MA104 cells with NeuGcGM3 being two- to three-fold more effective than NeuAcGM3 [75]. Furthermore, binding of triple-layered OSU to Lec-2 cells, which have 90% reduced sialylation of glycolipids and –proteins, is reconstituted to levels greater than that of control cells by addition of exogenous NeuGcGM3 [75]. In piglets, NeuGcGM3 rapidly declines in the first four weeks of life in the intestine while NeuAcGM3 is found in lower amounts and only gradually decreases over the first 16 weeks, suggesting NeuGcGM3 determines severity of disease [153]. Thus, while OSU can bind to multiple different gangliosides, GM3 is most likely the relevant attachment receptor in the porcine intestine.

**Figure 5.**
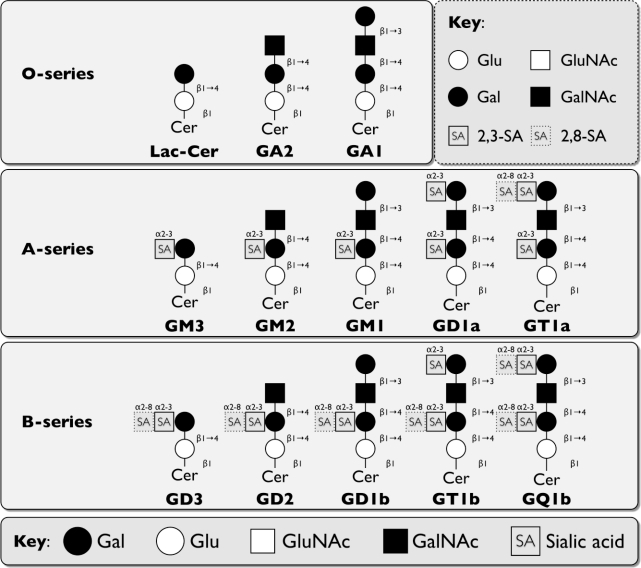
Schematic overview of O-, A- and B-series gangliosides discussed in text. Abbreviations: Glu: glucose, Gal: galactose, GluNAc: N-acetylglucosamine, GalNAc: N-acetylgalactosamine, Cer: ceramide, R: residue

More recently, binding of VP8* from the neuraminidase-sensitive porcine CRW-8 strain to GD1a was analyzed by STD-NMR spectroscopy [76]. This analysis indicated that the binding epitope for CRW-8 encompasses both the terminal and internal α2,3-linked sialic acid moieties of GD1a (Figure 5). The specificity of this interaction was confirmed by infectivity studies, in which pre-incubation with the homologous VP8* or Neu5Acα2Me but not a heterologous VP8* (from human Wa strain) decreased infection.

##### 2.3.1.2. Simian rotavirus (SA11 strain)

Binding of SA11 to neuraminidase-sensitive sialic acid moieties was recognized many years ago and shown to be responsible for the ability of this strain to hemagglutinate human or sheep erythrocytes [141]. Several subsequent studies have since then investigated the glycolipid ligands for the neuraminidase-sensitive strain SA11 with at times conflicting results. Preferential binding of SA11 to GA1 but not GM1, GD1a, or GT1b is observed by TLC, and GA1 coated polystyrene beads or anti-GA1 antibodies partially inhibit SA11 replication in MA104 cells [154]. In another study, SA11 bound to bovine brain material (primarily containing GM1, GD1a, GD1b, GT1b, Gd1b, some GD3, GT1a and traces of GQ1b) or isolated GM1 by Enzyme-linked immunosorbent assay (ELISA), and gangliosides from bovine brain material or isolated GM1 partially inhibited SA11 infection of monkey kidney LLC-MK2 cells in a dose-dependent manner [155]. Furthermore, the reduced susceptibility of neuraminidase-treated LLC-MK2 cells is partially restored by addition of bovine brain material or purified GM1. GA1 was not tested in this study. In a third study, glycolipids from the small intestine of Swiss Webster mice were separated by TLC and tested for binding to iodinated SA11 [156]. Characterization and comparison to synthetic glycolipids suggested binding to neutral GA1 and GA2, but not the sialic acid-containing derivatives GM1, GM2 (Figure 5). SA11 binding to GA1 was higher compared to GA2 in microtiter wells, and soluble GA1 was far superior to GA2 in inhibiting SA11 binding to adsorbed GA1 or GA2 in this assay. Due to the virus purification protocol, the contribution of double-layered or triple-layered particles to these binding profiles remains unclear. In addition, the relevance of the neuraminidase-dependent interaction of SA11 with these molecules during infection is unclear, as GA1, GA2 and GM1 have no neuraminidase-insensitive sialic acid groups (Figure 5), respectively. Therefore, the ability of CsCl-purified triple- but not double-layered particles from the neuraminidase-sensitive simian rotavirus SA11 to bind to 32 different gangliosides was tested by TLC [77]. Triple-layered SA11 particles bind to the terminal sialic acid residues of NeuGcGM3, sialylneolactotetraosylceramide (NeuAcα3Galβ4GlcNAcβ3Galβ4Glcβ1Cer), GM2 and GD1a. This suggests the minimal ganglioside binding epitope of SA11 is sialyl-galactose (NeuGc/NeuAcα3-Galβ) with a preference for N-glycolyl groups.

##### 2.3.1.3. Bovine rotavirus (NCVD, UK strain)

Bovine rotaviruses are important veterinary pathogens causing calf scour and include both neuraminidase-sensitive and -insensitive strains. Bovine rotavirus NCVD is a neuraminidase-sensitive strain that hemagglutinates erythrocytes and binds and infects MA104 cells [157]. In addition to SA11, Delorme *et al.* [77] also tested the ability of CsCl-purified triple-layered particles from the neuraminidase-sensitive NCVD and the neuraminidase-insensitive UK by TLC. Like SA11, NCVD and UK bind to sialylneolactotetraosylceramide (NeuAcα3Galβ4GlcNAcβ3Galβ4Glcβ1Cer), GM2 and GD1a but NCVD binds to the terminal sialic acid residues of NeuGcGM3 while UK binds to the internal sialic acid residues of NeuAcGM3 and GM1. Therefore, the proposed minimal ganglioside binding epitope for NCVD (like SA11) is sialyl-galactose (NeuGc/NeuAcα3-Galβ). The same minimal structure is found in all gangliosides recognized by UK (with a preference for NeuAc), but additional factors must play a role to explain the different ganglioside binding profile of UK. Interestingly, the main ganglioside found in the intestinal epithelium of two-day old calves is GM3 (NeuGc and NeuAc), with lower amounts of NeuGcGM2, NeuGcGM1 and NeuGcGD1a, while NeuGcGD2 (not bound by NCVD) is the major ganglioside in the adult bovine intestine [77]. Thus, NeuAcGM3 and NeuGcGM3 may represent the main attachment receptors *in vivo* for UK and NCVD, respectively.

##### 2.3.1.4. Rhesus rotavirus (RRV strain)

RRV binding to Caco-2 and MA104 cells in culture and intestines of mice is sensitive to neuraminidase treatment [137,158,159]. NMR spectroscopy demonstrates that the protease-resistant core of RRV VP8 binds α-anomeric N-acetylneuraminic acid with a *K_d_* of 1.2 mM independent of the α2-6 or α2-3 linkage in sialyllactose [78]. However, a strong preference for N-acetyl neuraminic acid (*K_d_* of 1.2 mM) was observed over N-glycolyl neuraminic acid (*K_d_* of 11 mM). A binding constant in a similar range (*K_d_* of 0.30 mM) was determined by isothermal titration calorimetry of RRV VP8* to Neu5Acα2Me [160].

X-ray crystallography of the RRV VP8* core bound to 2-*O*-methyl-α-d-*N*-acetyl neuraminic acid and NMR spectroscopy of the unliganded core demonstrated that the sialic acid binding site is located in an open-ended, shallow groove formed at one edge by two β-sheets [161]. No major structural changes are observed upon binding but local changes suggest a binding-induced fit of sialic acid in its recognition site. A similar structure (root mean square deviation less than 0.22 Å) was obtained when RRV and VP8* are crystallized at room temperature [160]. The VP8* core is a single globular domain of an 11-stranded anti-parallel β-sandwich that shares the same fold (but not the carbohydrate binding site) as the galectins (S-type lectins), which bind β-galactoside-containing oligosaccharides [161]. Similar to other viral sialic acid binding proteins (e.g., influenza hemagglutinin, polyomavirus VP1), interaction of hydrogen bonds from amino acids, water-mediated contacts, and van der Waals contacts with aromatic rings contribute to the interaction between protein and ligand. The key residues of VP8* that mediate contact directly with sialic acid’s glycerol chain and carboxylate group are R101 and S190, respectively. Mutation of R101 or S190 to alanine results in reduced sialic acid binding [160]. Wildtype, R101A, and S190A VP8* are able to compete with RRV infection of MA104 cells. However, mutant VP8* competition was less effective compared to wt VP8*. The wt and R101A mutant VP8* also inhibited RRV infection of neuraminidase-treated cells, leading to the suggestion that the sialic acid binding cleft also interacts with the aglycon portion of cell surface glycans.

#### 2.3.2. Neuraminidase-insensitive human rotaviruses (KUN, MO, DS-1, Wa strains)

Human rotavirus infection is typically not affected by neuraminidase treatment, nor do these strains hemagglutinate fresh erythrocytes [79,157]. The human rotaviruses KUN and MO strains infect MA104 cells in a neuraminidase-insensitive manner [79]. To identify the GSL required for infection, TLC and immunostaining were used to identify the GSLs expressed on MA104 cells, mainly the gangliosides GM1 and GM3 and the neutral GSL lactosylceramide (Figure 5). Of those, the sialic acid moiety of GM1 is insensitive to neuraminidase treatment. Addition of exogenous GM1 but not GA1 (*i.e.,* asialo-GM1) to MA104 cells inhibited infection with KUN and MO but not the neuraminidase-sensitive feline rotavirus strain FRV64. In addition, cholera toxin B subunit, which binds to GM1, is able to block infection with KUN and MO, but not FRV64. Hence, GM1 is a receptor for human KUN, MO strains in MA104 cells.

Metrizamide gradient purified (*i.e.,* preparation containing triple- and double-layered particles) human Wa and DS-1 rotavirus strains recognize GA1 but not GM1, GD1a, GT1b in TLC [154]. However, by STD-NMR spectroscopy VP8* of the strain Wa clearly shows binding to the internal α2,3-linked sialic acid moiety of GM1 and the branchpoint sugars (Gal, GalNAc) [76]. The specificity of this interaction was confirmed by infectivity studies, in which pre-incubation with the homologous VP8* but not heterologous VP8* (neuraminidase-sensitive porcine CRW-8 strain) or competition with cholera toxin B subunit (which specifically binds to GM1) decreased infection. Interestingly, Wa binding and infection was increased upon neuraminidase treatment, suggesting that removal of terminal sialic acids increases Wa receptor binding by exposing additional receptors, e.g., converting GD1a into GM1.

Analogous to RRV VP8*, the crystal structure of the VP8* core from the neuraminidase-independent human rotavirus DS-1 was also solved and shows the same galectin-like fold with a slightly wider groove than the sialic acid binding site in the RRV VP8* core [162]. In DS-1 the R101 is replaced by phenylalanine, which interacts with an adjacent β-sheet but does not contribute to any surface interactions. However, the adjacent extended binding region is highly conserved between the VP8* cores of RRV and DS-1 and capable of interacting with other more internal SA linkages [163].

### 2.4. Polyomaviridae

The *Polyomaviridae* family is a group of small double-stranded DNA viruses and derives its name from the ability of the viruses to form multiple tumors in mammals. All the members have a similar genome organization and capsid structure [164]. The ∼5kb circular genome forms a minichromosome with cellular histones and is encapsidated in an icosahedral virion [165–168]. The capsid is composed of 360 copies of the major capsid protein VP1, arranged in 72 pentamers linked by disulfide bonds and stabilized by calcium bridges [165,168]. Each pentamer interacts with a single copy of the minor capsid protein VP2 or VP3, which connects the viral DNA genome with the outer capsid shell [81]. VP1 is able to self-assemble into VLPs *in vitro* [169], and it contains the binding sites for the cellular receptors [170–172]. These viruses display a narrow host range and the interactions between the capsid and the receptors are an important determinant of viral tropism as reconstitution of certain non-permissive cells with the appropriate ganglioside can confer infectivity [83,173–175]. A number of receptors including gangliosides and glycoproteins are used by the polyomaviruses [176] (Table 1). Following the initial attachment to the cell surface, most polyomaviruses are internalized via either caveolin- or clathrin- mediated endocytosis [177–182]. Although they traffic through different endocytic compartments, all the polyomaviruses studied so far seem to converge in the ER, where viral capsid disassembly and membrane penetration are believed to occur [180,183–186]. There is also some recent evidence suggesting that the ganglioside receptors may govern the intracellular destination of these viruses [187]. In this section of the review, we will focus on the interactions between these viruses and various gangliosides.

#### 2.4.1. Murine Polyomavirus (MPyV)

MPyV was the first polyomavirus discovered (recovered from murine leukemia-causing extracts) [188] and was originally found to utilize sialic acid-containing receptors in the 1980s [189–191]. Treatment of human erythrocytes or murine 3T6 cells with neuraminidase that removes terminal sialic acids abolishes MPyV hemagglutination and prevents viral infection, respectively [190,191]. Subsequent restoration of the cells with α2,3-linked sialic acids is able to reconstitute these characteristics, suggesting a specific recognition of these sugar moieties [190,191]. Certain strains of MPyV are also able to bind to a second α2,6-linked, branching sialic acid [189]. The molecular determinant for binding to α2,3- or α2,6-linked sialic acid is a single-amino-acid difference in VP1 (residue 91 Glu *versus* Gly) that accounts not only for the receptor binding specificity difference but also changes in plaque size, hemagglutination properties, and tumor induction ability [192,193]. Crystal structures have shown that a shallow groove composed of several loops of VP1 on the virion surface accommodates these sialic acids [81,171]. These loops may be important in determining receptor specificity, as they display sequence variation among polyomaviruses [170,172]. The side chain of the Glu91 prevents the interactions with the carboxylate of the branched α2,6-linked sialic acid, thus explaining the differences in the second sialic acid binding [81]. Furthermore, biochemical studies demonstrated that MPyV has a low affinity for its receptors (with dissociation constants in the mM range) and these weak interactions are thought to result in more efficient release of the virion from the receptors, and therefore more rapid spread and increased pathogenicity [168,170]. This concept was further supported by the fact that the highly virulent LID strain of MPyV also has reduced virus-sialic acid interactions [174].

It was not until 2003 though when the true identity of the MPyV receptor was discovered [80]. Using a sucrose floatation assay, Tsai *et al.* demonstrated that the gangliosides GD1a and GT1b allow the virus to float to the lighter fractions in sucrose gradients [80]. Furthermore, supplementation of non-permissive rat or mouse cells with GD1a can restore MPyV infectivity [80,194]. The terminal α2,3-linked sialic acid linked to the Gal sugar moiety of GD1a and GT1b are most likely in contact with VP1, based on modeling of these two gangliosides onto the VP1-oligosaccharide crystal structure [80] (Figure 5). The binding pocket for the α2,6-linked sialic acid in the crystal structure does not seem to interact with these gangliosides; instead, it may be involved in binding to glycoproteins [80,81]. Consistent with this, the glycoprotein α4β1 integrin has been suggested as a co-receptor for MPyV at a post-attachment level [82,83]. Addition of GD1a to rat C6 glioma cells or murine NIH 3T3 cells increases the co-localization between the ER and MPyV, and facilitates a productive infection [80,187]. Coating an artificial particle with GD1a is sufficient to deliver it to the ER, suggesting that ganglioside binding may serve as a general ER targeting signal [187]. Whether this also applies to other polyomaviruses binding to their respective gangliosides, remains to be determined.

#### 2.4.2. Simian Virus 40 (SV40)

SV40 was identified as a contaminant of cell cultures that were used for poliovirus vaccine production [195]. It turns out, however, to have been an invaluable tool to study many fundamental questions in molecular biology [164]. Whether SV40 is a human pathogen remains controversial [196]. Early studies suggested that SV40 did not recognize sialic acids [197], but this was later disproven. The ganglioside GM1 was identified to be the cellular receptor for SV40 at the same time that the MPyV receptors were identified [80]. Similar to the MPyV-receptor interactions, the binding of GM1 to SV40 is relatively weak [172]. A high resolution crystal structure obtained from SV40 VP1 pentamers, with or without a GM1-derived oligosaccharide, reveals that there are no major conformational changes in VP1 upon GM1 binding [172]. VP1 binds to both the internal α2,3-sialic acid and Gal-β1,3-GalNAc of GM1 (Figure 5) in a shallow groove, which may explain the strict specificity of SV40 for GM1 [172]. Although both SV40 and MPyV bind to α2,3-linked sialic acids, the binding occurs in different orientations and conformations for these two viruses [172]. A carbohydrate microarray screen identified that the simian-derived N-glycolyl GM1 ganglioside [GM1(Gc)] is a better receptor for SV40 than GM1 [198]. The differences may lie in the higher affinity of a deeper pocket formed by two externalized loops of VP1 for the additional hydrophilic hydroxyl group present in GM1(Gc) [172, 198]. During attachment to cells SV40 also interacts with its co-receptor, the class I major histocompatibility proteins to enter cells in a caveolin-dependent or -independent manner [84,85,181,199,200].

The interaction between SV40 and GM1 is a dynamic process. Kukura *et al.* developed a methodology that combines interferometric scattering detection with single-molecule microscopy to track single quantum dot-labeled SV40 VLPs on supported lipid bilayers [201]. They observed both sliding and tumbling motions of VLPs, suggesting a rapid exchange between the VP1 pentamers and the GM1 receptors. They also observed back and forth rocking motions between distinct nanodomains when GM1 was present at a high concentration, which presumably results from the existence of small GM1 aggregates [201]. After the initial interaction of SV40 with GM1, invagination of the membrane is required for successful entry. A recent study showed that the binding of SV40 particles or VP1 pentamers to GM1 alone is sufficient to induce membrane curvatures that can proceed to form membrane tubules [202]. Using the murine cell line GM95, deficient in glucose-based glycolipids and supplemented with various GM1 species, the authors demonstrated that the length of the acyl chain of the lipid moiety and not the ceramide base of GM1 is crucial for the endocytosis and the establishment of a productive infection of SV40 [202]. This formation of the membrane curvatures is caveolin-independent. A similar observation has also been made with MPyV [202]. These data led to the proposal that the receptor binding sites present in the pentamers can induce lipid clustering and invagination and eventually lead to caveolin-independent endocytosis [202].

#### 2.4.3. BK virus (BKV) and JC virus (JCV)

BKV and JCV are two well-established human polyomaviruses. They were identified in the same year and were both named after the initials of the patients from whom they were isolated [203,204]. BKV is the causative agent of polyomavirus-associated nephropathy in renal transplant patients and of hemorrhagic cystitis in bone marrow transplant recipients, whereas JCV is known to be associated with progressive multifocal leukoencephalopathy, a demyelinating central nervous system disease in immunosuppressed individuals [205]. Using a sucrose floatation assay *in vitro*, BKV was found to bind to gangliosides GD1b and GT1b [86]. Compared to GM1, both GD1b and GT1b carry an additional α2,8-linked sialic acid motif that may be uniquely recognized by BKV [86] (Figure 5). Addition of these gangliosides to non-permissive cells confers susceptibility to BKV infection *in vivo* [86]. There is also some conflicting evidence regarding the usage of sialylated glycoproteins as cellular receptors by BKV. Restoration of sialylation on N-linked glycoproteins in sialic acid-stripped cells restores BKV infectivity [87]. Treatment with proteinase K, however, does not affect BKV interaction with membranes [86]. The ganglioside usage of JCV in cells is not well characterized but there is limited evidence pointing to the involvement of GT1b in JCV infection in susceptible human neuroblastoma IMR-32 cells [88]. By TLC, JCV VLPs bind to lactosylceramide, GM3, GD2, GD3, GD1b, GT1b, GQ1b, and weakly to GD1a but not to GM1a, GM2, or galactocerebroside [88,206] (Figure 5). An N-linked glycoprotein with α2,6-linked sialic acids and the serotonin receptor 5HT2aR serve as receptors for JCV in glial cells [89–91].

#### 2.4.4. Merkel Cell Polyomavirus (MCPyV)

MCPyV is a newly discovered polyomavirus from Merkel cell carcinomas (MCC), an aggressive form of skin cancer [207]. The viral DNA was found in a high frequency of MCC tumors, suggesting a causal relationship between MCPyV and MCC [205]. Although no infectious virion has been isolated for MCPyV, the ganglioside GT1b was suggested to be a cellular receptor based on experiments performed with MCPyV VP1 capsomeres [92]. The fact that only GT1b, but not GD1a or GD1b, confers binding to MCPyV VP1 indicates that both the α2,3-linked terminal and α2,8-linked internal sialic acids of GT1b are required for the interaction [92] (Figure 5).

### 2.5. Parvoviridae

The *Parvoviridae* family contains small icosahedral viruses with a single-stranded DNA genome of approximately 5 kb [208]. The genome is flanked by terminal repeats required for DNA replication, encodes one or two nonstructural proteins (NS1, NS2) required during viral replication, and 2–3 capsid proteins (VP1, VP2 and sometimes VP3), which form the viral capsid. Replication occurs in the nucleus. The *Parvovirida*e family is divided into two subfamilies, the *Parvovirinae* and *Densovirinae*, which infect mammalian and invertebrate hosts, respectively. The former is further subdivided into five genera, of which members of the genera *Dependovirus* (bovine adeno-associated virus, BAAV) and *Erythrovirus* (human parvovirus B19, simian parvovirus) will be discussed here because they interact with gangliosides and globosides, respectively.

#### 2.5.1. Erythroviruses

Erythroviruses belong to the autonomous parvoviruses [208]. These viruses are species-specific and have a narrow host range for hematopoietic progenitor cells. They are replication competent but do not induce cell division and thus only infect cells going through S phase that have the required host cell component to initiate DNA synthesis. Their genome encodes two structural proteins, VP1 and VP2, and the nonstructural protein NS1, with the inherent ability of VP2 to self-assemble into VLPs.

##### 2.5.1.1. Human parvovirus B19

Human Parvovirus B19 was identified in 1975 in serum from infected patients (for review see [209]). It is the causative agent of erythema infectiosum (Fifth disease) as well as anemia, arthropathy (joint disease), and fetal loss. Transmission occurs through the respiratory tract, by blood transfusion or *in utero*. B19 binds to erythroid precursors and their destruction accounts for the anemia associated with B19 infections in patients with high red cell turnover. One of the receptors utilized on the erythrocyte surface by B19 is globoside. However, while globoside is necessary for B19 binding, it is not sufficient for virus entry into cells [95]. The co-receptor required for viral entry of erythrocyte precursors is α5β1 integrin [95]. Thus, in case of mature red blood cells, which highly express globoside but not α5β1 integrin, B19 can bind to but not enter these cells [95]. In addition to erythrocytes, B19 can also infect other immune cells like T cells, B cells, macrophages and follicular dendritic cells [210]. On non-erythrocyte cells, the autoantigen Ku80 functions as a co-receptor to mediate binding and entry of B19 [96]. While globoside was the first receptor for B19 to be identified, it is not the only one utilized. Further studies are required to fully delineate the repertoire of cell surface molecules and sequence of events required during viral entry.

**Figure 6.**
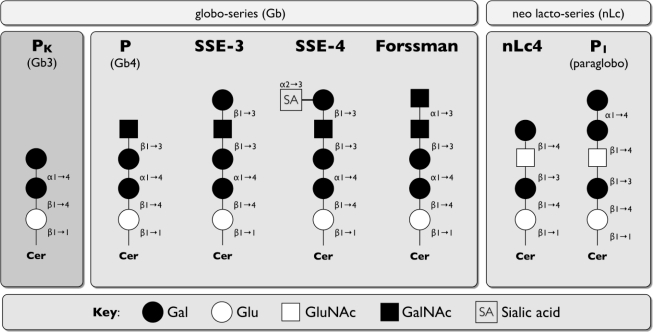
Comparison of discussed carbohydrate structures used during attachment of parvovirus B19. Schematic representation of nLC and Gb attachment receptors in comparison to the non-binding globoside Gb3. Abbreviations: Glu: glucose, Gal: galactose, GluNAc: N-acetylglucosamine, GalNAc: N-acetylgalactosamine, Cer: ceramide, R: residue

For the purpose of this review, we will focus only on globoside, also called blood group P antigen, because it is a neutral GSL in the globo-series [7]. The blood group P antigen system contains three major antigens P_1_, P (both common), and P^k^ (rare). P is the globoside Gb4 (globotetraosylceramide), P^k^ is Gb3 (globotriaosylceramide), and P_1_ is an α4Gal-structure on the paragloboside structure [7] (Figure 6). Globoside was identified as the cellular receptor for B19 through a series of biochemical and competition experiments. B19 VLP could hemagglutinate red blood cells from individuals with the P_1_ phenotype (carry P and P_1_ antigens) and P_2_ phenotype (P antigen) but not p phenotype (lack all three antigens) [93], and only individuals with the rare p phenotype (Galβ1,4Glc-Cer) are not infected with B19 [211]. Biochemical analysis of B19 VLP binding to purified GSLs by TLC demonstrated binding to globoside Gb4 and Forssmann antigen but not P^k^ [93] (Figure 6). Furthermore, B19 infection of late erythroid-colony forming units (CFU-E) was blocked by either competition with globoside or an anti-globoside monoclonal antibody [93]. Subsequent analysis of additional neutral GSL from multiple tissues identified additional weak binding of B19 VLPs binding to two globoseries GSLs (SSEA-3, SSEA-4) and nLc4 (neolactotetraglycosylceramide) [94] (Figure 6), which are part of the same biosynthetic pathway as P antigen [7]. This indicates the minimal carbohydrate binding epitope may be the GluNAc/GalNAcβ1,3Gal sequence shared between all [94] (Figure 6). Cryo-electron microscopy studies of B19 VLPs with globoside followed by computer modeling indicate that the globoside receptor likely binds to the hydrophobic depressions at the icosahedral three-fold axis of the viral capsid as a trimer [212].

##### 2.5.1.2. Simian parvovirus (SPV)

SPV is closely related to B19 (for review see [213]). It was identified in 1992 in cynomolgus macaques (*Macaca fascicularis*) co-infected with immunosuppressive type D simian retrovirus. Experimental infection of cynomolgus macaques shows similar symptoms to B19 infections in humans, *i.e.* transient anemia (decrease in red blood cells) and reticulocytopenia (decrease in immature red blood cells), decreased erythroid and myeloid lineages in the bone marrow, and hydrops fetalis (accumulation of fluid in fetal compartments) and fetal death in pregnant animals. Like B19, SPV also uses globoside on the erythrocyte precursors and is able to infect human bone marrow mononuclear cells [97]. Specifically, SPV VP2 VLPs were shown to hemagglutinate human erythrocytes. Hemagglutination was blocked by globoside and Forssmann antigen, which differ by an additional GalNAc group in the latter (Figure 6), but not other GSL (lactosylceramide, ceramide trihexoside, asialoganglioside GM2). Whether SVP can bind to additional neutral GSL like B19 remains to be determined.

#### 2.5.2. Dependoviruses

Adeno-associated viruses (AAVs) are nonpathogenic viruses that depend on a helper virus (mostly adenovirus or herpesvirus) for replication [208]. AAVs latently infect their hosts by integrating into the host DNA while lytic infection and production of infectious virus occurs only in the presence of a helper virus. AAVs are typically detected as contaminants of viral stocks from primate, human, avian, bovine, ovine, or equine origin. Recombinant AAVs are promising vectors for gene therapy because of their nonpathogenic nature, broad tropism, ability to transduce dividing and non-dividing cells and ability for long-term expression *in vivo* [214]. The diverse tropisms of AAVs are due to differences in receptor usage. However, the only AAV shown to date to require GSL for infection is bovine AAV (BAAV) [98].

BAAV like the related AAV4 and AAV5 requires sialic acid during infection [98]. BAAV transduction of cells was reduced by treatment with several exoneuraminidases, including the neuraminidase from *S. pneumoniae*, which is specific for α2-3 linked sialic acid, and blocked by competition with wheat germ agglutinin, a sialic acid-binding lectin [98]. Interestingly, binding of BAAV in contrast to AAV5 was not blocked by removal of terminal sialic acids. BAAV binding and transduction were protease resistant, and transduction and DNA uptake but not binding were blocked by glycosylceramide synthase inhibitors. Incorporation of gangliosides but not neutral GSLs into GSL-depleted COS or ganglioside-deficient C6 cells was able to restore transduction. These data demonstrate that BAAV requires gangliosides for transduction, probably at the level of virus internalization [98]. However, the specific ganglioside(s) mediating this step in the viral life cycle remains to be identified.

## 3. Conclusions

GSLs are a group of molecules with a large carbohydrate head group and a lipid tail, which inserts into the cell membrane. Due to many different possible modifications in both the sugar and the lipid portion, these molecules exhibit great diversity that is not only cell type- and tissue-dependent, but also changes during differentiation, activation of cells, as well as during ontogenic development and hematopoiesis. Many viruses take advantage of these specific patterns on the cell surface for recognition of target cells. For example, expression of specific GSLs during individual stages of development is a likely reason for the observed age-dependent disease manifestation of rotaviruses in young animals or older animals for RHDV. Conversely, the lack of expression of a given GSL can result in natural resistance to a given viral disease, e.g., inability of B19 to infect individuals of the p phenotype that lack globoside. Furthermore, a given glycan repertoire on the cell surface can have a profound impact on viral evolution and epidemiology. For example, individual norovirus strains may only infect a subset of the population, but through slight changes in the binding site different strains cover a wide range of binding profiles so that any individual in the population is at risk of being infected. Receptor switching and changing antigenicity then allows the virus to successfully escape the host immune system and expand into naive populations. In addition, the typically weak binding affinities of GSL and their ligands makes them ideal candidates for attachment receptors that concentrate viruses (and other microbes) near the cell surface to enable interaction with additional receptor molecules that facilitate internalization. Virus interaction with a GSL typically occurs via the carbohydrate portion, while the lipid portion, in case GSLs serve as viral entry receptors, controls the intracellular fate of these viruses. Thus, for most virus families discussed in this review (calici-, rota-, and parvoviruses), GSLs merely serve as attachment receptors that facilitate binding to the cell surface but not endocytosis. Endocytosis of these viruses depends on the interactions between the virus and other receptor molecules. However, for viruses that utilize GSLs as an entry receptor, such as the polyomaviruses discussed herein, the intracellular trafficking of the virus is governed by specific virus-GSL interactions. In the case of MPyV, for example, the entry receptor GD1a targets the virus to the ER [187]. The fact that all polyomavirus studied so far travel to the ER despite interacting with various gangliosides displaying differing sugar moieties suggests that the lipid portion of the GSL is important for controlling the intracellular transport of the viruses. In fact, SV40 endocytosis is governed by the acyl chain length of GSLs [202]. The driving force for endocytosis may be a virus-induced clustering of GSLs leading to formation of membrane tubules/invaginations as each individual capsid protein within a repeating subunit (*i.e*., SV40 VP1 pentamer) binds to GM1 [202]. Similarly, interaction of Shiga toxin B subunit, which is active only in its pentameric form, with its receptor Gb3 induces narrow tubular membrane invaginations [215]. While membrane invaginations can clearly form within model membranes in both cases [202,215], further work is needed to determine whether GSL clustering alone or clustering induced recruitment of proteins is required for targeted trafficking of the respective ligand.

In summary, GSL-virus interactions are a common feature of viruses from multiple different virus families and represent an attractive anti-viral target. However, the varying intricacies of such interactions need to be taken into account when designing GSL-mimics that can effectively block virus binding to host cells.

## Figures and Tables

**Table 1.** Summary of viruses and their glycosphingolipid receptors discussed in the text and respective references.

**VIRUS**	**GLYCOSPHINGOLIPID RECEPTORS**	**OTHER RECEPTORS**	**REFERENCE**
***Caliciviridae***			
*Norovirus*			
Human Norovirus (HuNoV)	Histo-blood group antigens (HBGA) on type 1, 2, and 3 GSLs	Heparan sulfate, sialic acid on SLe^x^	[44,61–64]
Murine Norovirus (MNV)	Terminal α2,3-linked sialic acid on GD1a and GT1b		[70] and this article
Bovine Norovirus (BoNoV)	αGal of HBGA		[71]
*Lagovirus*			
Rabbit Hemorrhagic Disease Virus (RHDV)	A and H type 2 HBGA		[72]
***Reoviridae***			
*Rotavirus*		Integrins α2β1, α4β1, αvβ3, αxβ2, hsc70	[73]
*Neuraminidase-sensitive*			
Porcine Rotavirus: OSU strain	Sialic acid on GM3		[74,75]
Porcine Rotavirus: CRW-8 strain	Terminal and internal α2,3-linked sialic acid on GD1a		[76]
Simian Rotavirus: SA11 strain	Sialyl-galactose on NeuGcGM3, sialylneolactotetraosylceramide, GM2, and GD1a		[77]
Bovine Rotavirus: NCVD strain	Sialyl-galactose (NeuGc/NeuAcα3-Galβ) on NeuGcGM3, sialylneolactotetraosylceramide, GM2, and GD1a		[77]
Rhesus Rotavirus: RRV strain	N-acetyl neuraminic acid		[78]
*Neuraminidase-insensitive*			
Bovine Rotavirus: UK strain	Sialyl-galactose (NeuAc) on NeuGcGM3, GM1, GD1a, GM2, sialylneolactotetraosylceramide		[77]
Human Rotavirus: KU, MO, DS-1, Wa strains	GM1		[76,79]
***Polyomaviridae***			
Murine Polyomavirus (MPyV)	Terminal α2,3-linked sialic acid on GD1a and GT1b	Integrin α4β1	[80–83]
Simian Virus 40 (SV40)	GM1	Class I MHC	[80,84,85]
BK Virus (BKV)	α2,8-linked disialic acid on GD1b and GT1b	Unknown glycoprotein	[86,87]
JC Virus (JCV)	Terminal α2,3-linked sialic acid on GT1b	Serotonin receptor 5HT2aR; Terminal α2,6-linked sialic acid on an unknown glycoprotein	[88–91]
Merkel Cell Polyomavirus (MCPyV)	Terminal α2,3-linked sialic acid and α2,8-linked disialic acid on GT1b		[92]
***Parvoviridae***			
*Erythrovirus*			
Human Parvovirus B19	HexNAcβ1,3Gal on globoside Gb4 (P antigen), SSEA-3, SSEA-4, and nLc4	Integrin α5β1, autoantigen Ku80	[93–96]
Simian Parvovirus	Globoside and Forssmann antigen		[97]
*Dependovirus*			
Bovine Adeno-associated Virus (BAAV)	Unknown ganglioside		[98]

**Table 2.** Disaccharide precursors of glycoconjugates [6,67].

**Name**	**Structure**	**Residue (R)**
Type 1	Galβ1-3, GlcNAcβ1-R	N-,O-glycoproteins, GSLs of the lactoseries (Lc)
Type 2	Galβ1-4, GlcNAcβ1-R	N-,O-glycoproteins, GSLs of the neolactoseries (nLc)
Type 3	Galβ1-3, GalNAcα1-R	O-glycoproteins (core 1), GSLs of the ganglioseries (Gg)
Type 4	Galβ1-3, GalNAcβ1-R	GSLs of the globoseries (Gb)
